# Optically Transparent Gold Nanoparticles for DSSC Counter-Electrode: An Electrochemical Characterization

**DOI:** 10.3390/molecules27134178

**Published:** 2022-06-29

**Authors:** Jessica Barichello, Donatella Spadaro, Sara Gullace, Alessandro Sinopoli, Pietro Calandra, Alessia Irrera, Fabio Matteocci, Giuseppe Calogero, Stefano Caramori, Carlo Alberto Bignozzi

**Affiliations:** 1 IPCF-CNR, Istituto per i Processi Chimico-Fisici, Viale F. Stagno d’Alcontres 37, 98158 Messina, Italy; jessica.barichello@uniroma2.it (J.B.); donatella.spadaro@cnr.it (D.S.); alessia.irrera@cnr.it (A.I.); 2 CHOSE—Center for Hybrid and Organic Solar Energy, Department of Electronic Engineering, University of Rome “Tor Vergata”, 00133 Rome, Italy; fabio.matteocci@uniroma2.it; 3 ISIS UMR 7006, CNRS, Université de Strasbourg, 8 Allée Gaspard Monge, 67000 Strasbourg, France; gullace@unistra.fr; 4 QEERI—Qatar Environment & Energy Research Institute, Hamad Bin Khalifa University, Doha P.O. Box 34110, Qatar; asinopoli@hbku.edu.qa; 5 CNR-ISMN, National Research Council—Institute for the Study of Nanostructured Materials, Via Salaria km 29.300, Monterotondo, 00015 Rome, Italy; pietro.calandra@cnr.it; 6 Department of Chemical and Pharmaceutical Sciences, University of Ferrara, Via L. Borsari 46, 44121 Ferrara, Italy; carloalberto.bignozzi@unife.it

**Keywords:** dye-sensitized solar cell, gold electrode, transparent PV, Z907, cobalt electrolyte, nanoparticle

## Abstract

A gold nanoparticles transparent electrode was realized by chemical reduction. This work aims to compare the transparent gold nanoparticles electrode with a more commonly utilized gold-film-coated electrode in order to investigate its potential use as counter-electrode (CE) in dye-sensitized solar cells (DSSCs). A series of DSSC devices, utilizing I^−^/I^3−^ and Co(III)/(II) polypyridine redox mediators [Co(dtb)3]^3+^/^2+^; dtb = 4,4′ditert-butyl-2,2′-bipyridine)], were evaluated. The investigation focused firstly on the structural characterization of the deposited gold layers and then on the electrochemical study. The novelty of the work is the realization of a gold nanoparticles CE that reached 80% of average visible transmittance. We finally examined the performance of the transparent gold nanoparticles CE in DSSC devices. A maximum power conversion efficiency (PCE) of 4.56% was obtained with a commercial I^−^/I^3−^-based electrolyte, while a maximum 3.1% of PCE was obtained with the homemade Co-based electrolyte.

## 1. Introduction

**Figure 1:**
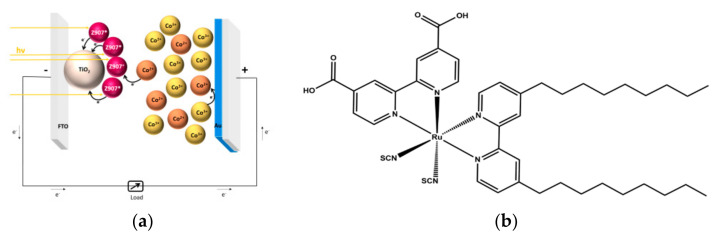
(**a**) Cross-section schematic view of the investigated DSSC. (**b**) Molecular structure of the dye sensitizer Z907 utilized in this study.

Dye-sensitized solar cell (DSSC) is a well-known photovoltaic (PV) technology due to its potential features of transparency, color tunability, low dependence from the solar incident angle and easy fabrication process [1,2,3] over Si-based solar cells. The working system was inspired by photosynthesis and the dye works, as chlorophyll does, harvesting photons [4]. The photo-anode, the electrolyte and the counter-electrode (CE) are the three main characters present in a DSSC device. The anchored dye on a photo-anode has the role of catching photons from the sunlight and transferring them in the semiconductor, usually through the carboxylic moiety. Electrons flow in the external circuit, reaching the cathode. The redox couple, generally composed of I^−^/I^3−^ in the electrolytic solution, regenerates the oxidized dye. Electrons from the external circuit, passing through the catalyst on the cathode, regenerate the redox couple and, in this way, the cycle is closed and it can continue (Figure 1a). In recent years, researchers in PV technologies have been attracted by the uncommon characteristics of transparency [1,2], bifaciality [5,6], the working capacity under a low sunlight intensity and the potential inclusion of friendly components [7,8,9,10] that can ensure applications in building-integrated photovoltaic (BIPV), indoor environments [11] and greenhouses [12,13,14]. Transparency is ensured by the combination of different factors within the device: a low semiconductor thickness, the choice of the wavelength’s dye absorption, the transparency of the electrolyte and that of the cathode. The high transparency in the PV cell penalizes the power conversion efficiency (PCE) [15]. The DSSC devices showed impressive stability under accelerated tests over a large area with a robust module fabrication process [14], but the interaction between platinum and iodide/iodine is not the best solution for long-term stability [16]. The I^−^/I^3−^ redox couple has several issues, such as competition with the dye for the visible light absorption and the formation of ion pairs between I^3−^ and oxidized dye resulting in enhanced electron recombination. On the other hand, Pt-based CEs are rare and expensive [17]. Therefore, the substitution of platinum with other, more stable catalyst became an active field of research [18,19,20]. The development of new electrolytes and catalysts with high transmittance allows for the esthetic characteristics of DSSC to be exploited, promoting them as ideal candidates for indoor application and BIPV. Recently, the possibility of using gold-based electrodes in association with iodine-free electrolytes has been reported for both dye-sensitized solar cells and quantum-dot-sensitized solar cells [21,22]. Indeed, as widely known, gold is not stable in iodide-based solutions. Looking at the different iodine-free electrolytes, copper- [23,24] and cobalt- [25,26] based electrolytes showed better PV performances, while other metal-based electrolytes are still under investigation [27]. The presence of gold in DSSC has been widely studied in the photo-anode components [28,29], but few research articles consider gold as a counter-electrode [30]. Besides the mandatory characteristics of high conductivity and high catalytic activity, CEs must have high reflectivity and high optical transmittance to be exploited in a bifacial DSSC device and in BIPV. Furthermore, the need to implement transparent counter electrodes is urgent for other PV technologies. For the most recently reported perovskite solar cells (PSC), a gold layer is utilized as the most efficient top electrode, but its opacity does not allow for the development of transparent PSC devices and their use in BIPV [31,32,33,34]. The aim of this work is to develop an efficient and transparent cathode, with gold nanoparticles deposited by chemical reduction.

In literature, one of the most common methods to deposit a gold film is thermal evaporation [20]. Recently, the laser ablation technique has been investigated to develop semi-transparent gold nanoparticles CE with excellent results in terms of PCE [30]. In this work, we aim to fabricate, for the first time, to the best of our knowledge, a semi-transparent gold nanoparticles CE by chemical reduction: this is a new strategy, which has never been used in DSSC. This process can reach the semi-transparency of the deposited gold layer; moreover, it is safer for the human operator, as it uses a less dangerous precursor, than thermal decomposition. We avoided the presence of chromium, usually used to ensure the good adhesion of the gold layer to the FTO glass in the thermal evaporation technique. Our research is worth investigation, since the chemical deposition process of a semitransparent gold nanoparticle layer may be used in semitransparent perovskite solar cells as the top electrode instead of opaque gold or other metals. In this work, we firstly compared the deposition of gold nanoparticles by chemical deposition and the more commonly employed technique, thermal evaporation, to deposit a continuous film of gold. Deep characterizations, such as scanning electron microscopy (SEM) and atomic force microscopy (AFM), were utilized to investigate the structural surfaces of the deposited layer. Then, we compared the catalysts in DSSC devices composed using Z907 as a sensitizer (Figure 1b) and a Co-based electrolyte, performing characterizations such as electrochemical impedance spectroscopy, transmittance spectroscopy and a preliminary current density–voltage (J-V) scan. In the second part of the work, we focused on the device’s fabrication, to test finally the new catalyst (gold nanoparticles obtained by chemical reduction) with two electrolytes, a commercial reference I^−^/I^3−^ electrolyte and our homemade Co-based electrolyte, comparing our results with those in the literature.

## 2. Results and Discussion

### 2.1. SEM and AFM Measurements of Counter-Electrodes

**Figure 2:**
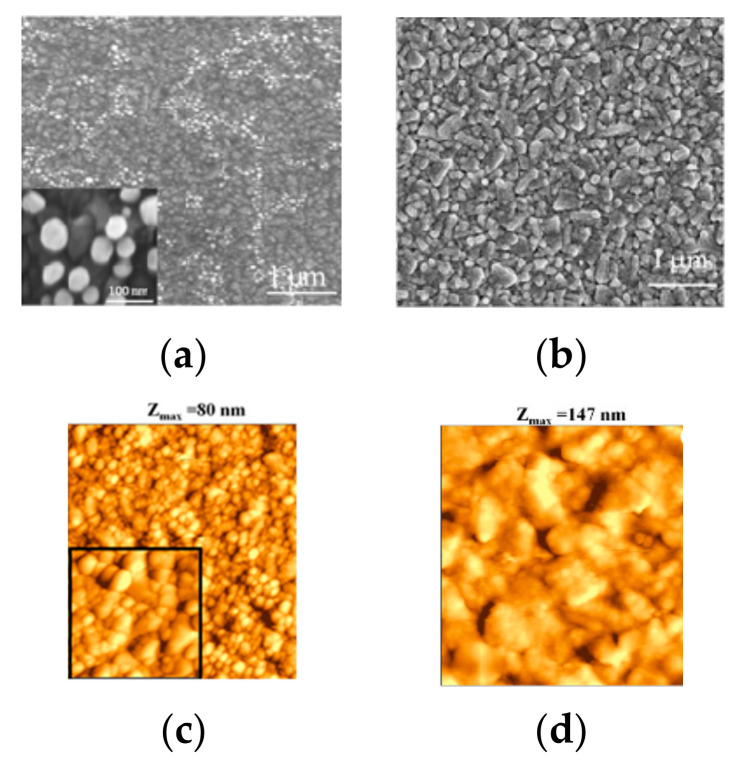
(**a**,**b**) Plan-view SEM image of (**a**) gold nanoparticles counter-electrode deposited by chemical reduction, and (**b**) gold film counter-electrode deposited by thermal evaporation. (**c**,**d**) 2 μm × 2 μm AFM images of the samples deposited by (**c**) chemical reduction, (**d**) thermal evaporation. In (**c**) the inset is 0.5 μm × 0.5 μm AFM scan of the corresponding sample.

**Figure 3:**
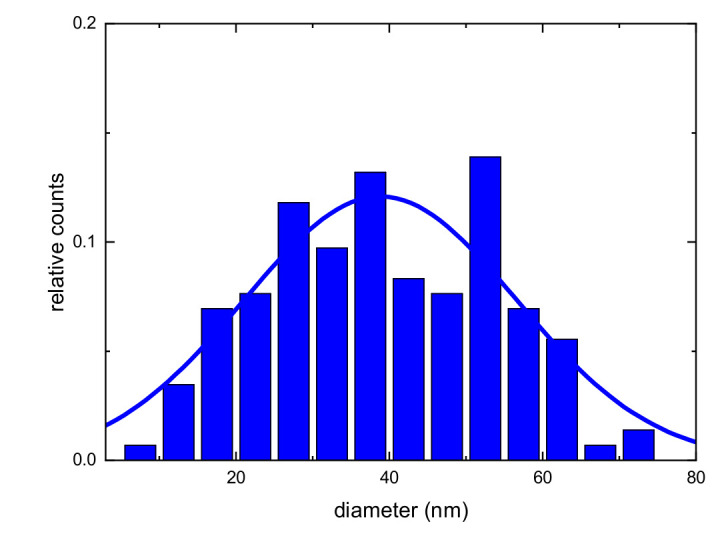
Distribution of the diameters D of the Au nanoparticles in the samples deposited by chemical reduction. The continuous line is the Gaussian fits of the experimental data.

We performed a detailed study of the structural properties of the different counter-electrodes using scanning electron microscopy (SEM) and atomic force microscopy (AFM). In Figure 2, the plan-view SEM and AFM images of the different samples are shown. From the SEM and AFM images in Figure 2a,c, we can clearly observe that the Au nanoparticles (obtained by chemical reduction) do not form a continuous film on the substrate. On the other hand, in Figure 2b–d, the continuous film obtained with Au, deposited by thermal evaporation, is clearly visible. Au nanoparticles decorate the large grains of the FTO constituting the substrate. Using SEM analyses, we can find the diameter D of the Au nanoparticles in the sample, deposited by chemical reduction. In Figure 3, the diameter distribution of nanoparticles is reported. In particular, it is observed that the diameter of the gold nanoparticles obtained by chemical reduction is about 39 ± 16 nm. The density of gold nanoparticles obtained by chemical reduction is 8.3 × 10^9^ cm^−2^, as can be seen in the SEM and AFM images (Figure 2a–c).

By means of the AFM, we can directly evaluate the roughness ⌠ of the samples. A computed value of average roughness is the root–mean–square variation (i.e., standard deviation) ⌠ of the surface height profile from the mean height. We obtained the roughness σ for each sample by the corresponding AFM images using the SPMLab7 software. The σ value for each sample was calculated by averaging the values obtained by at least five AFM images. Following this procedure, we obtain very similar <⌠> values for all the samples (with gold nanoparticles and with Au films), of approximately 10 nm, suggesting that the roughness of the samples with gold nanoparticle or Au film is determined by the roughness of the underlying substrate (FTO).

**Figure 4:**
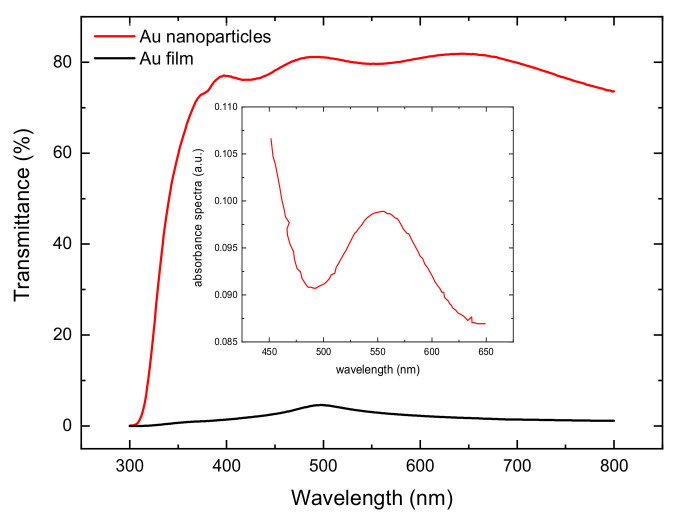
Transmittance spectra: Au nanoparticles (red) and Au film (black). Inset. Absorption spectra of the visible plasmon band of Au nanoparticles on the FTO surface.

A further characterization that we conducted on our gold catalyst is transmittance spectroscopy. The gold film deposited by thermal evaporation showed no transmittance, while Au nanoparticles’ CE obtained by chemical reduction reached a high transmittance value of 80% from 380 to 780 nm (Figure 4). Furthermore, the absorbance spectra in the inset in Figure 4 clearly show the plasmonic nature of the Au nanoparticles deposited by chemical reduction. Results from transmittance measurements concerning the Au nanoparticle cathode confirm the transparency feature of our CE.

### 2.2. Electrochemical Study

For the electrochemical study, we tested both the dummy cells (cathode–cathode) and DSSC. The fabrication process of the cells is described in Section 3, as well as the components of our homemade Co-based electrolyte.

**Figure 5:**
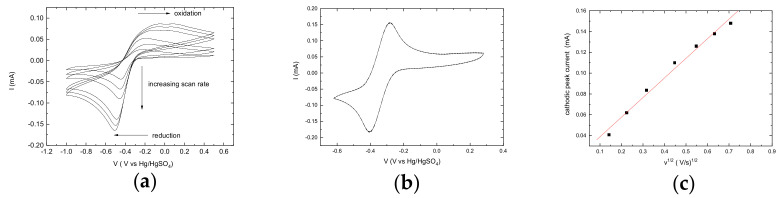
(**a**) CV of 1.675 × 10^−3^ M Co(II)/(III) at Au nanoparticles electrode (by chemical reduction) in ACN/0.1 M LiClO_4_. (**b**) CV of Co(II)/(III) ~2 × 10^−3^ M at an Au film (by thermal evaporation) working electrode. (**c**) Cathodic peak current vs. v1/2.

The Au nanoparticles obtained by the chemical reduction counter-electrode display a reasonable catalytic activity when compared to Au films obtained by thermal evaporation counter-electrodes whose are considered the top-performing counter-electrodes for Co(II)/(III) mediators at present [30,35]. For deposited Au nanoparticles, the cyclic voltammogram of a 1.675 × 10^−3^ M equimolar Co(II)/(III) solution in CAN (Figure 5a,b) displays the typical quasi-reversible behavior of the Co(DTB)^3+/2+^ couple with a peak separation of 270 mV at 100 mV. The peak separation exhibits a marked dependence on the scan rate, ranging from ~270 mV at slow scan rates (20–100 mV/s) to ~450 mV at a scan speed of 500 mV/s. The voltammogram shows a marked asymmetry in the anodic and cathodic branch: while the oxidation gives rise to a broad and hill-defined peak, Co(III) reduction appears as a sharp and well-defined feature indicative of a transfer coefficient greater than 0.5. The cathodic peak current shows a linear dependence on $\sqrt{v}$, as expected from a diffusion-controlled process (Figure 5c). From the slope of the I vs. $\sqrt{v}$ curve, by using the reported diffusion coefficient in diluted acetonitrile solutions (6.7 10^−6^ cm^2^/s) for Co(III)(DTB)_3_^3+^ an electroactive area of 0.17 cm^2^ is obtained, in reasonable agreement with the geometrical area (0.18 cm^2^). This is expected, since the electroactive gold nanoparticles do not cover the entire FTO surface, which is essentially inactive for the redox chemistry of cobalt.

**Figure 6:**
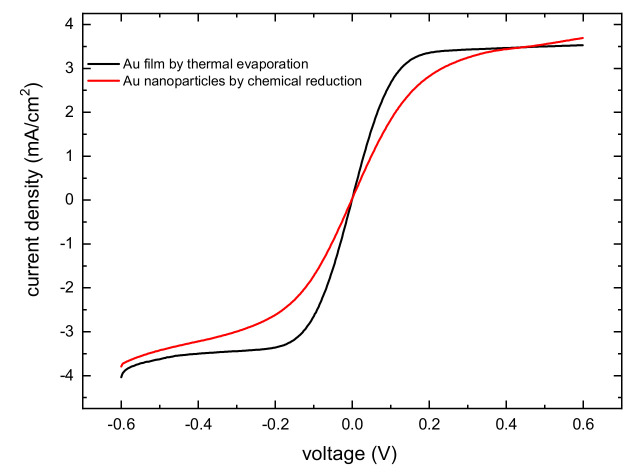
Linear scan voltammetry of 0.67 M Co(II)/(III) in a symmetric thin-layer cell (electrode spacing ca. 100 µm) made with either Au nanoparticles or evaporated gold electrodes.

However, gold films display a more reversible behavior (Figure 5b at 100 mV/s), with a substantial wave symmetry and a smaller peak separation, which varies much less pronouncedly with the scan speed (122 mV at 100 mV/s, 172 mV at 500 mV/s). The linear scan voltammetry (Figure 6) (1 mV/s) in a symmetrical two-electrode thin-layer cell (spacing ~100 μm, the same employed for DSSC assembly) confirms the superior performance of the gold film, which shows a significantly higher slope (0.033 A/V) than the particle-based CE (0.019 A/V). In both cases, the limiting current, determined by the mass transport of the redox couple is very similar ~4 mA/cm^2^ and is met at very low over-potentials. This suggests that the main limitation of the Co(II)/(III) couple arises from the diffusional transport, which makes it difficult to access a purely kinetic-controlled regime at which the linear (low field) approximation of the Butler–Volmer equation could be used.

**Figure 7:**
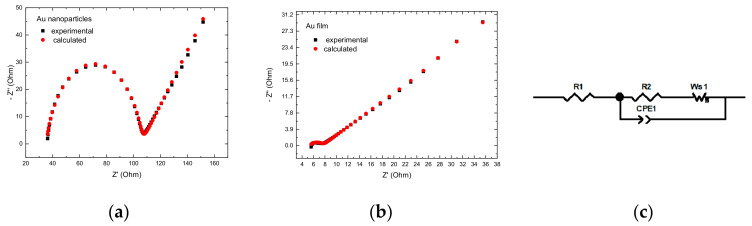
Nyquist plots for symmetrical thin layer cell. Electrode Spacing ~100 μm: (**a**) Au nanoparticles; (**b**) Au film; (**c**) equivalent circuit for the symmetric cells.

For this reason, electrochemical impedance spectroscopy (EIS) measurements (Figure 7) performed on the same cells at equilibrium potentials is useful for discriminating the time domains of the electron transfer and mass transport processes. Figure 7a reveals a relatively simple picture, with a kinetic loop at high frequencies (modelled by a parallel RC element, where charge transfer resistance was associated with a non-ideal interfacial capacitance described by a constant phase element (CPE)), determined by the electron transfer, followed by a Warburg behavior. The charge transfer resistance at the nanoparticle electrodes is 350 Ω/cm^2^ (Figure 7a), whereas, at the gold film (Figure 7b), the charge transfer is much smaller, appearing as a small, depressed semicircle at 18.4 KHz with Rct = 7.5 Ω/cm^2^ followed by the straight line typical of the diffusional impedance. The equivalent circuit for fitting the experimental data is shown in Figure 7c where, according to the Kirchhoff laws for two identical interfaces, R2 = 2Rct and CPE1 = C/2. A short Warburg element (Ws1) accounts for the diffusional resistance of the redox couple in the thin-layer cell.

**Figure 8:**
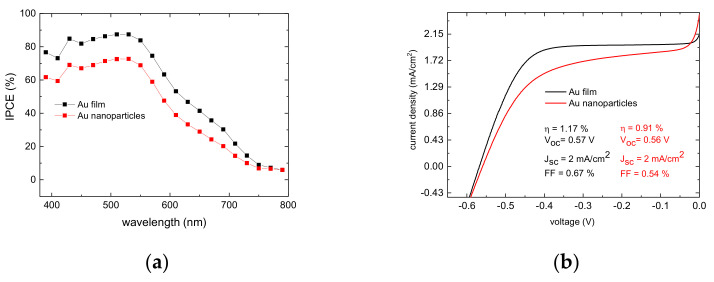

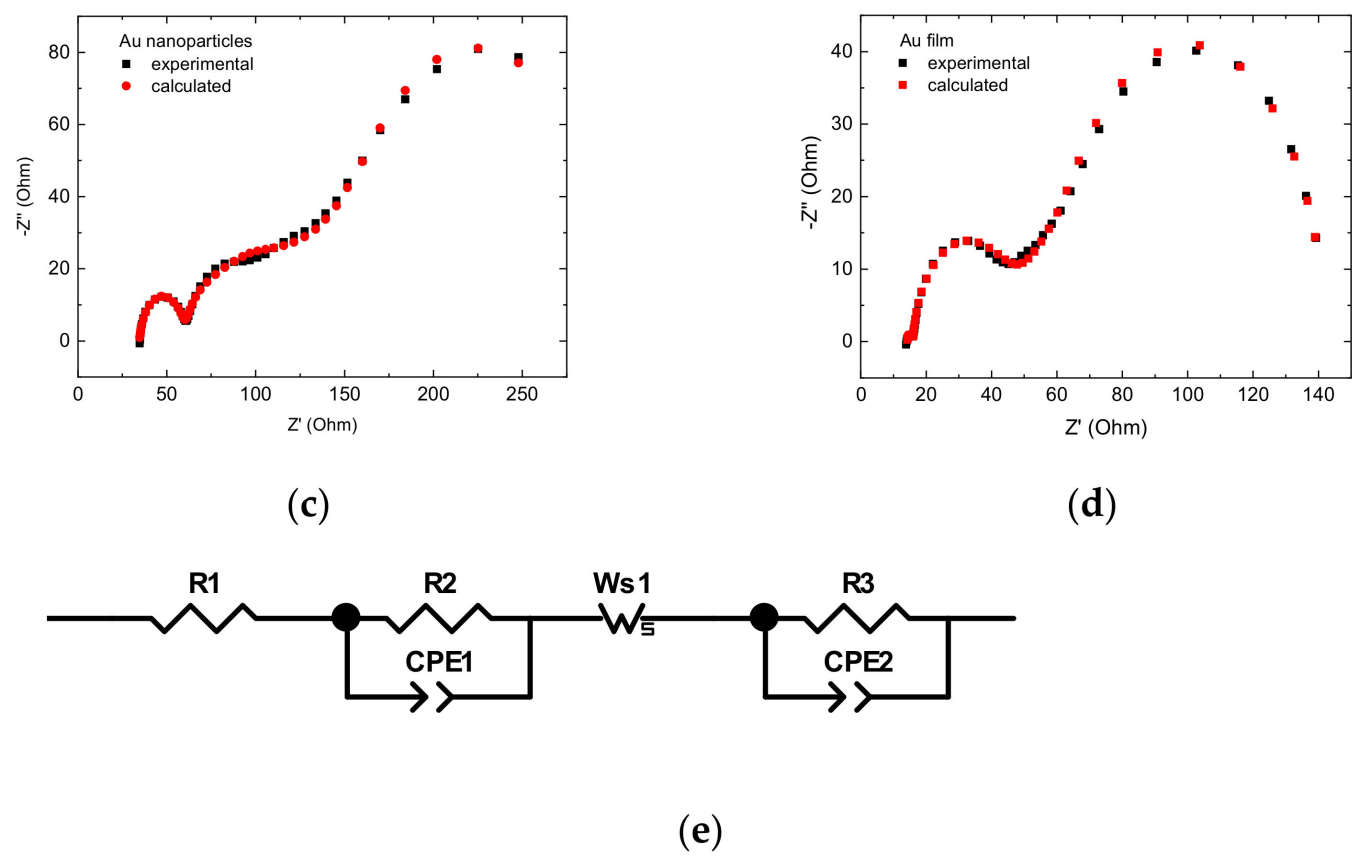
(**a**) Photo-action spectra in the presence of the two different catalytic CEs: Au film (black) and Au nanoparticles (red) (**b**) Representative current density–voltage (J-V) curves in the presence of the two different CEs: nanoparticles (red); film (black) (**c**,**d**) Complex plane plots of Cobalt mediated DSSC at Voc in the presence of two different types of counter-electrodes: Au nanoparticles/FTO (**c**) and Au film (**d**). (**e**) Equivalent circuit used to fit the EIS response of the DSSCs at Voc.

The analysis of the DSSC performance completes the electrochemical picture of the properties of these new counter electrodes. The photo-action spectra (Figure 8a) under low monochromatic light intensity shows satisfactory maximum quantum yields, in the order of 65% and 80% for the Au nanoparticle and Au film, respectively. The DSSC performance under 0.067 W/cm^2^ illumination (Figure 8b) is consistent with the preliminary electrochemical study. Both the Jsc and the Voc of the cells equipped with the gold film and with the gold nanoparticles CEs are very similar with a Jsc~2 mA/cm^2^ and a Voc of 0.56 V. The main difference is found in the fill factor which, for the Au nanoparticles (0.54) is significantly lower than that found for the Au film (0.67), due to the higher serial resistance determined by a sluggish electron transfer. The current voltage characteristics of DSSCs have been shown to follow the diode equation according to (1) [36]:

(1) $$I=I_{\mathrm{photo}}-I_{0}\;(\exp(\frac{e\;\left(V+IRs\right)}{nKT})-1)-\frac{V+IRs}{Rsh}$$

where I is the total current produced by the device at a given voltage Va. I_photo_ is directly proportional to absorbed photon flux. I_0_ is the dark current that flows through the non-ideal diode (ideality factor n) at the applied forward voltage Va = (V + IRs) where Rs is the sum of the series resistance in the cell provided by contact resistance (RΩ), charge transfer (R_CT_) and transport (RT) resistances and diffusional resistance (RD), according to

Rs = RΩ + RT (TiO_2_)+ R_CT_ (TiO_2_) + R_CT_ (CE) + RD (2)

The effect of Rs on the JV curve becomes important under strong direct polarization, i.e., when the dark current becomes significant and visibly affects the descending branch of the J/V when one moves from the plateau photocurrent to Voc. It is one of the major factors that affects the Fill Factor (FF = (V_max∙J_max)/(V_OC_∙J_SC_)) [8]. A large Rs leads to a proportionally large potential loss across these parasitic resistances, and thus decreases the maximum power point of the cell. On the other hand, Va/Rsh accounts for charge leaks via short circuits in the cell, the typical reaction of electrons with the redox couple at the ohmic contacts, and is most visible at low forward voltages, where the diode dark conductivity is low.

The lower FF found with the gold nanoparticles electrode has repercussions for the cell efficiency, which is about 22% lower than that of the analogue cells based on evaporated gold film. EIS spectroscopy (Figure 8c,d) at Voc provides a simultaneous picture of the main charge transfer and charge transport processes in the operational cell. The electric equivalent of the DSSC at Voc is reported as Figure 8e. When the cell is kept at Voc, electron accumulation inside TiO_2_ occurs, increasing the semiconductor conductivity. Therefore, the transport resistance of the TiO_2_ film RT(TiO)_2_) < R_CT_ (TiO_2_) and the transmission line model can be simplified with the circuit based on two parallel RC elements schematized in Figure 8e [37]. As expected, by moving from higher to lower frequencies, three loops can easily be identified. At higher frequencies, the EIS picture is dominated by the charge transfer impedance at the counter electrode (R2-CPE1 mesh), followed at middle frequencies by the impedance of the TiO_2_/electrolyte interface (R3-CPE2 mesh), which, under Voc, can be simplified as another parallel circuit. The lower-frequency arc is characteristic of the mass transport within the mesoporous TiO_2_ phase, and is modeled by a Nernst impedance (3) [38].

(3) $$Z=\frac{1}{Y_{0}\sqrt{j\varpi }}\;\tan hB\;\sqrt{j\varpi }$$

where *B* =$\;\frac{\delta }{\sqrt{D}}$.

It can be clearly seen that the latter dominates, since, in both cases, its width is ≥100 Ω. From the *B* term, obtained from the fitting and assuming a film thickness of ~6 microns, one can obtain the apparent diffusion coefficient of the Co(II)/(III) couple, of the order of 5 × 10^−8^ and at least 10–20 times smaller than that of the I^3−^ ion in viscous liquids. While a charge transfer loop of the CE based on gold nanoparticles is clearly evident, with a Rct = 86 Ω/cm^2^, the catalytic activity of the evaporated Au film is so high that the charge transfer loop is barely observable, with an Rct ~8 Ω/cm^2^, in agreement with the results found with the symmetrical cell experiments.

### 2.3. Optimized Photovoltaic Parameters

**Figure 9:**
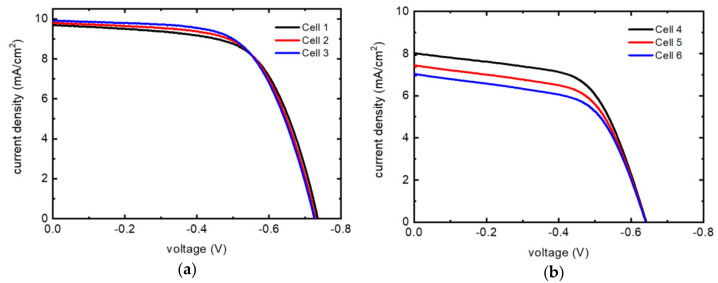
Current density–voltage (J-V) curves at scan rate: 20 mV/s. (**a**) HSE electrolyte (**b**) Home-made Co-based electrolyte.

On the basis of our results, we optimized the device assembly process (see Section 3.3) to test the J-V performances of the gold nanoparticles CE deposited by chemical reduction. The aim is to increase the device performances in order to compare the Au nanoparticle-based CE with the references already present in the literature. We focused on comparing the gold nanoparticles CE with a commercial electrolyte high-stability electrolyte (HSE), based on iodine/triiodide couple, and a homemade Co-based electrolyte. The Co-based electrolyte is worthy of investigation due to the compromised stability caused by the iodine presence in the most common electrolytes in the literature. In Figure 9, current density–voltage (J-V) curves of the optimized DSSC with gold-nanoparticle CEs are reported.

In a recent work [39], in a Z907-based DSSC cell assembled with a reference platinum cathode and a reference commercial I^−^/I^3−^ based electrolyte, similar values of PCE (4.64 and 4.35%) compared to our study (≈4.55%) were obtained (Table 1). Comparing PCE results with the state-of-art in the literature (Table 1), obtained under similar conditions in terms of dye and electrolyte, is important to benchmark the efficiency of the working system of our transparent gold nanoparticles CE obtained by chemical reduction with the reference platinum CE. The main deviation of the literature data compared to the present investigation involves the current density, spanning from 10.34 to 12.41 mA/cm^2^ in [39] and averaging 9.8 mA/cm^2^ in our devices. This is understandable if we consider that, when aiming to realize a transparent cathode, the deposited gold nanoparticles do not form a continuous Au film (Figure 2a), such as the one we obtained by thermal evaporation method, which, conversely, had really low transmittance (Figure 4). This partly leads to a loss in catalyst efficiency: since non-covered FTO grains are inactive in the electrolyte regeneration process. Another factor arises from the lack of back reflection from our highly transparent counter electrodes, which reduces the overall light-harvesting efficiency of the solar cell. Nevertheless, the applications of transparent DSSCs are much more promising than those of opaque devices for architectural integration. The open-circuit voltages and the FF present higher values in our devices. This is a consequence of the fabrication process: in this work, a blocking layer was deposited to suppress the back electron transfer processes (i.e., increase the shunt resistance Rsh of Equation (1)), while, in [39], this step has not been performed. However, the highest reported PCE in the literature for Z907 dye is in [40], with a value of 6.8%. The main difference is found in the current density due to the previously discussed reasons. In this case, FF is similar to ours due to the use of the same fabrication process.

Once the efficiency of the transparent cathode was confirmed with the use of a reference electrolyte as an HSE, and once comparisons in the literature were found, we tested the gold clusters’ transparent cathode with a Co-based electrolyte.

Devices with a Co-based electrolyte showed a loss of efficiency compared to the iodide-based electrolyte, due to mass transport limitations arising from the larger size of the Cobalt complexes based on sterically hindered ligands. The slow diffusion of the cobalt mediator decreased by constrictive channel effects within the mesoporous TiO_2_ [41]. Moreover, Co(II)/(III) lacks the fast regeneration observed for I^−^/I^3−^: in the latter case, the rate of dye regeneration was boosted by the electrostatic attraction between TiO_2_ intercalated Li^+^ and iodide, enhancing the local surface concentration of this anionic redox couple [42]. On the other hand, this favorable effect was absent with positively charged Co(II) mediators. This also contributes to explaining the lower PV conversion efficiency observed with the cobalt-based electrolyte, resulting in a PCE decreased by a ca. 305 factor with respect to an equivalent iodide-mediated cell.

## 3. Materials and Methods

### 3.1. Synthesis of Gold Nanoparticles and Au Film

Gold nanoparticles’ synthesis was performed by phase-transfer catalyst reaction: an aqueous solution of HAuCl_4_, (3 mM, 3 mL) was mixed with a (Tetraoctylammonium bromide) TOAB/toluene solution (5 mM, 8 mL) and the resulting mixture was kept under vigorous stirring for two hours. In this step, the HAuCl_4_ was transferred from water to the organic phase. The Au(III) to Au(0) reduction was then performed by adding, drop by drop, an aqueous solution of NaBH_4_ (0.04 M, 3 mL) to the same mixture under continuous stirring. Although the reaction mixture turned gradually but quickly to purple, indicating fast Au(0) formation, it was kept at ambient conditions to ensure the completion of the reaction; after that, the organic phase was separated. This method was expected to allow for the preparation of golden nanoparticles in the range 5–6 nm [43]. To avoid post-synthesis ageing effects or nanoparticle growth, a suitable amount of dodecylmercaptane (2 µL per 10 mL of Au(0)-containing solution), which is a well-known capping agent, was then added to the organic solution. Au(0) nanoparticles’ deposition onto FTO glass was performed by casting, placing 60 mL of solution onto 2 cm × 2 cm squared FTO pieces, allowing for solvent evaporation and calcinating them at 350 °C for one hour. It is worth noting that the addition of dodecylmercaptane to the Au(0)-containing solution was necessary to avoid nanoclusters agglomeration during Au(0) nanoparticles’ deposition onto FTO glass and to achieve the formation of a uniform Au(0) nanostructured film. An FTO substrate was located in the vacuum chamber of the evaporator and a 2-nm-thick gold layer was deposited on top of them at 480 °C by electron beam evaporation using high-purity (99.9%) gold pellets as a source. The pressure during the evaporation was about 1 × 10^−7^ mbar. The filament current of the gun was 270 mA. The deposition system was a modified Varian physical vapor deposition (PVD) chamber. The system was equipped with an electron gun evaporation source, 3 crucibles with linear motion and a 3-cm broad-beam ion source. The vacuum was maintained by ion pumps and turbo pumps. The deposition rate and film thickness were monitored in situ by a quartz microbalance. The substrate temperature can be varied from room temperature to about 700 °C. Au film counter electrodes were fabricated by the thermal evaporation of gold (~70 nm) over a 7-nm chromium adhesion layer, thermally evaporated onto well-cleaned FTO glass.

### 3.2. Device Fabrication

A Z907 standard solution was prepared by dissolving 20 mg of complex in 50 mL of ethanol. The photoanode was prepared by depositing a film of the semiconductor paste (Dyesol 18NRT) on the FTO-conducting glass pre-treated with TiCl_4_ solution (blocking layer). Two edges of the FTO glass plate were covered with four layers of adhesive tape (3 M Magic) to control the thickness of the film and to mask electric contact strips; the TiO_2_ paste was uniformly spread on the substrate by sliding a glass rod along the tape spacer. The resulting mesoscopic oxide film was estimated to be around 10 μm thick and transparent, showing negligible light scattering. The active area of the obtained photo anodes was approximately 0.181 cm^2^. After drying the TiO_2_, the covered glass plates were sintered in air for 30 min at 450 °C, cooled to about 80 °C and soaked for one night in the dye solution; excess dye was removed by rinsing with ethanol and dried in oven. Cell assembly for the first preliminary electrochemical studies was carried out by making a sandwich structure where a parafilm frame of about 100 µm was placed between the two cathode electrodes for dummy cells and between the cathode and anode for DSSC. The homemade cobalt electrolyte was placed in the device through drop-casting and the cell structure was held together by a clothespin. To test the J–V performances of the gold nanoparticles deposited by chemical reduction, we focused on improving a device’s fabrication. The latest optimized DSSCs were sealed using a 25-µm-thick Surlyn frame, as described in [6], and a thermal press. Commercial HSE electrolyte was used as a reference electrolyte to test the gold nanoparticles’ cathode. Concerning the homemade, Co-based electrolyte, the composition was as follows: 0.18 M Co(DTB)_3_(ClO4)_2_; 0.028 M Co(DTB)_3_(ClO_4_)_3_; 0.1 M LiClO_4_; 0.2 M TBP in acetonitrile solvent. DTB = 4,4′-di-tert-butyl-2,2′-bipyridine. TBP = 4,4′-tert-butyl-2,2′-bipyridine.

### 3.3. Instruments and Materials

AFM images were obtained in tapping mode using a MultiMode Nanoscope IIIa (Digital Instruments, Chapel Hill, NC, USA). The device was equipped with a J scanner, which was calibrated using the manufacturer’s grating. Ultrasharp tips (Noncontact “Golden” Silicon cantilevers, NSG10S, typical force constant 11.5 N/m, resonant frequency 255 kHz) were used. Height images were flattened to remove background slopes. No other filtering procedures were performed on AFM images. SEM characterization was performed by a Zeiss Supra 25 Scanning Electron Microscope (SEM) with a Schottky field emission gun. The acceleration voltage can be varied in the 0.1–30 kV range. A Gatan Digital Microscope Software was used for the statistical analysis. The conductive glass plates (FTO glass, fluorine-doped SnO_2_, sheet resistance 15 Ω/cm^2^) and the titanium oxide (TiO_2_) nanopowder (<25 nm) were purchased from Solaronix SA and Sigma-Aldrich, respectively. All Solvents and chemicals employed were of reagent or spectrophotometric grade and were used as received. The synthetic dye cis-RuLL′(SCN)_2_ (L = 4,4′-dicarboxylic acid-2,2′-bipyridine, L′ = 4,4′-dinonyl-2,2′-bipyridine), called Z907, was purchased from Dyesol. EIS for cells and DSSCs was carried out at open-circuit voltage by using a sinusoidal 10-mV perturbation in the 10^−5^–10^−2^ frequency range. Fitting was achieved with ZsimpWin. Linear sweep voltammetry was carried out at 5 mV/s. All electro-chemical measurements were carried out with an Eco Chemie PGSTAT 302/N workstation.

## 4. Conclusions

The present study investigated the chemical deposition of gold nanoparticles and compared this to the most-studied Au film deposition by thermal evaporation with the aim of developing a transparent and efficient counter-electrode for DSSC devices. Furthermore, from this investigation, we improved the understanding of the structure–property relationships of nanomaterials and molecules. We showed an impressive transparency of 80% of the gold-nanoparticles’ CE and a deep characterization of structural features was carried out. We finally utilized the gold nanoparticle transparent cathode in a DSSC device and, when compared to the literature, we obtained similar results under the same electrolyte conditions.

Moreover, we extended our investigation to test this counter-electrode with a Co-based electrolyte, since it is widely known from the literature that metal-based electrolytes are more sustainable for this kind of device. The maximum PCE obtained with a transparent gold cathode and a Co-based electrolyte was 3.1%: this is a satisfactory result considering the steric hindrance of the Cobalt complexes in the electrolyte.

The development of a transparent counter-electrode by chemical deposition opens room not only for DSSC applications, but also for other PV technologies, such as perovskite solar cells, where the use of gold as top electrode is widespread but the reflectivity of the layer does not allow for the fabrication of a real, semi-transparent device. Nanochemistry and nanoplasmonics could play an important role in future PV technologies.

## Figures and Tables

**Table 1:** Comparison of the PV parameters of the cells with gold nanoparticles’ CEs with two different electrolytes (HSE and homemade, Co-based, this study) and platinum CE in the literature.

Cell	Catalyst	Electrolyte	Jsc (mA/cm^2^)	Voc (V)	FF (%)	PCE (%)
1	Gold nanoparticles	HSE	9.7	0.73	64	4.53
2			9.79	0.73	64	4.54
3			9.9	0.73	63	4.56
[39]	Platinum	AN50	12.41	0.7	53	4.64
		Hi30	10.34	0.66	63	4.35
[40]		I^−^/I^3−^	13.6	0.72	69	6.8
4	Gold nanoparticles	Homemade Co-based	8.01	0.64	60	3.1
5			7.45	0.64	59	2.83
6			7.03	0.64	59	2.66

## Data Availability

Not applicable.
